# Engineering Expression Cassette of *pgdS* for Efficient Production of Poly-γ-Glutamic Acids With Specific Molecular Weights in *Bacillus licheniformis*

**DOI:** 10.3389/fbioe.2020.00728

**Published:** 2020-07-09

**Authors:** Dong Wang, Huan Wang, Yangyang Zhan, Yong Xu, Jie Deng, Jiangang Chen, Dongbo Cai, Qin Wang, Feng Sheng, Shouwen Chen

**Affiliations:** ^1^State Key Laboratory of Biocatalysis and Enzyme Engineering, Environmental Microbial Technology Center of Hubei Province, College of Life Science, Hubei University, Wuhan, China; ^2^Wuhan Junan Biotechnology Co., Ltd., Wuhan, China

**Keywords:** *Bacillus licheniformis*, Poly-γ-glutamic acid, molecular weight, PgdS depolymerase, controllable degradation

## Abstract

Poly-γ-glutamic acid (γ-PGA) is an emerging biopolymer with various applications and γ-PGAs with different molecular weights exhibit distinctive properties. However, studies on the controllable molecular weights of biopolymers are limited. The purpose of this study is to achieve production of γ-PGAs with a wide range of molecular weights through manipulating the expression of γ-PGA depolymerase (PgdS) in *Bacillus licheniformis* WX-02. Firstly, the expression and secretion of PgdS were regulated through engineering its expression elements (four promoters and eight signal peptides), which generated γ-PGAs with molecular weights ranging from 6.82 × 10^4^ to 1.78 × 10^6^ Da. Subsequently, through combination of promoters with signal peptides, the production of γ-PGAs with a specific molecular weight could be efficiently obtained. Interestingly, the γ-PGA yield increased with the reduced molecular weight in flask cultures (Pearson correlation coefficient of −0.968, *P* < 0.01). Finally, in batch fermentation, the highest yield of γ-PGA with a weight-average molecular weight of 7.80 × 10^4^ Da reached 39.13 g/L under glutamate-free medium. Collectively, we developed an efficient strategy for one-step production of γ-PGAs with specific molecular weights, which have potential application for industrial production of desirable γ-PGAs.

## Introduction

Poly-γ-glutamic acid (γ-PGA), an anionic polymer composed of repeated D- and L-glutamic acid units via γ-amide linkages, is mainly produced by *Bacillus* species (e.g., *B. licheniformis*, *B. subtilis*, *B. amyloliquefaciens*) (Sirisansaneeyakul et al., 2017). Due to its versatile physical properties, γ-PGA has been used in various fields, including agriculture, food, cosmetics, and pharmaceutical industries (Cao et al., 2018). Notably, depending on the producers, the molecular weight (Mw) of γ-PGA varied with a range from 1.0 × 10^4^ to over 2.0 × 10^6^ Da (Sirisansaneeyakul et al., 2017). The γ-PGAs with different Mws are exploited in different applications (Ogunleye et al., 2015). For example, γ-PGAs with high-Mws are used as superior flocculants in the wastewater treatment (Yokoi et al., 1996). The medium-Mw γ-PGAs (∼9.9 × 10^5^ Da) can efficiently remove basic dyes from solution (Inbaraj et al., 2006). Low-Mw γ-PGAs (2.0 × 10^4^–2.7 × 10^5^ Da) can be used as the drug carrier and tissue engineering nanocomposite in the biomedical industry (Shu et al., 2014). Thus, the controlled Mw is critical for the development and application of γ-PGA.

Previously, efforts have been made to change the Mws of γ-PGAs through changing fermentation conditions (Zeng et al., 2016; Feng et al., 2017). Also, several physical methods such as ultrasonication and heating (Pérez−Camero et al., 1999), as well as chemical methods like acidic and alkaline hydrolysis (Kubota et al., 1996), have been applied to depolymerize γ-PGA. However, since those methods lack precise control of Mws, the increasing polydispersity leads to a complicated purification process, which in turn limits the application of γ-PGA. By contrast, the enzymatic depolymerization via γ-PGA depolymerase is an alternative way to produce γ-PGA with desirable Mws with several superiorities, including mild reaction conditions and non-pollution (Yao et al., 2009). However, the extraction and purification of the depolymerase PgdS and subsequent hydrolysis processes are time-consuming and comparatively tedious, hampering the application of the enzymatic method for tailor-made γ-PGA production.

Recently, one-step fermentation is becoming an attractive method for biorefinery. For example, γ-PGA with a wider range of Mws (4.0 × 10^4^–8.5 × 10^6^ Da) were achieved in *B. subtilis* via expression of different γ-PGA synthetases (Halmschlag et al., 2019). Several endo-type PgdS depolymerases have been characterized (Suzuki and Tahara, 2003; Tian et al., 2014; Sha et al., 2018). Tian et al. (2014) characterized the PgdS hydrolase from *B. licheniformis* WX-02 and expressed this enzyme to achieve efficient production of low-Mw γ-PGAs. Moreover, Sha et al. (2018) achieved production of γ-PGA with different Mws through expressing four PgdS hydrolases with different hydrolytic activity. These results indicate that Mws of γ-PGA are correlated with PgdS hydrolase activity.

In this study, we develop a simple system for one-step production of γ-PGA through regulating the expression of PgdS depolymerase in *B. licheniformis* WX-02, a glutamate independent producer. The promoter and signal peptide are the vital factors for expression and secretion of proteins (Zhang et al., 2016). The Mws of γ-PGA was systematically modified by manipulating the promoters and signal peptides independently and in combination. By generating PgdS with different hydrolytic activities, γ-PGAs with a broad range of Mws were efficiently produced. Therefore, the strategy of engineering PgdS expression cassette is feasible to produce γ-PGAs with specific Mws.

## Materials and Methods

### Strains, Plasmids and Culture Conditions

All strains and plasmids employed in this work were listed in Table 1. The pHY-300PLK plasmid and temperature-sensitive plasmid T_2_(2)-ori were used to construct the expression and deletion vectors, respectively. All strains were generally cultivated at 37°C in Luria Bertani (LB) medium. When necessary, antibiotics (20 μg/L kanamycin, or 20 μg/L tetracycline) were supplemented into the media.

**TABLE 1:** Strains and plasmids in this study.

Strains or plasmids	Relevant properties	References
**Escherichia coli**		
DH5α	F^–^, φ80d*lac*ZΔM1, Δ(*lacZYA-argF*)U169, *deoR*, *recA*1, *endA*1, *hsdR*17 (rk^–^, mk^+^), *phoA*, *supE*44, λ^–^*thi*-1, *gyrA*96, *relA*1	Stored in lab
**Bacillus licheniformis**		
WX-02	Wide-type (CCTCC M208065)	Wei et al., 2010
WX-02Δ*pgdS*	deletion of *pgdS* in WX-02	This study
**Control**		
SP01	WX-02Δ*pgdS* derivative with expression plasmid pHY300PLK	This study
**Signal peptide**		
SP11	WX-02Δ*pgdS* derivative with expression plasmid pHYP43-SPsacC	This study
SP12	WX-02Δ*pgdS* derivative with expression plasmid pHYP43-SPvpr	This study
SP13	WX-02Δ*pgdS* derivative with expression plasmid pHYP43- SPbprA	This study
SP14	WX-02Δ*pgdS* derivative with expression plasmid pHYP43-SPggt	This study
SP15	WX-02Δ*pgdS* derivative with expression plasmid pHYP43-SPaprE	This study
SP16	WX-02Δ*pgdS* derivative with expression plasmid pHYP43-SPpgdS	This study
SP17	WX-02Δ*pgdS* derivative with expression plasmid pHYP43-SPyvpA	This study
SP18	WX-02Δ*pgdS* derivative with expression plasmid pHYP43-SPsacB	This study
**Promoter**		
SP23	WX-02Δ*pgdS* derivative with expression plasmid pHYPpgdS-SPbprA	This study
SP33	WX-02Δ*pgdS* derivative with expression plasmid pHYPbacA-SPbprA	This study
SP43	WX-02Δ*pgdS* derivative with expression plasmid pHYPbprA-SPbprA	This study
**Combination**		
SP21	WX-02Δ*pgdS* derivative with expression plasmid pHYPpgdS-SPsacC	This study
SP25	WX-02Δ*pgdS* derivative with expression plasmid pHYPpgdS-SPaprE	This study
SP28	WX-02Δ*pgdS* derivative with expression plasmid pHYPpgdS-SPsacB	This study
SP31	WX-02Δ*pgdS* derivative with expression plasmid pHYPbacA-SPsacC	This study
SP35	WX-02Δ*pgdS* derivative with expression plasmid pHYPbacA-SPaprE	This study
SP38	WX-02Δ*pgdS* derivative with expression plasmid pHYPbacA-SPsacB	This study
SP41	WX-02Δ*pgdS* derivative with expression plasmid pHYPbprA-SPsacC	This study
SP45	WX-02Δ*pgdS* derivative with expression plasmid pHYPbprA-SPaprE	This study
SP48	WX-02Δ*pgdS* derivative with expression plasmid pHYPbprA-SPsacB	This study
**Plasmids**		
T_2_(2)-ori	*E. coli* and *B. subtilis* shuttle vector; Ori_*pUC*_/Ori_ts_, Kan^r^	Qiu et al., 2014
T_2_-*pgdS*	T_2_ derivation with deletion fragment of *pgdS*	This study
pHY300PLK	*E. coli*-*Bacillus* shuttle vector; Amp^r^ in *E. coli*,Tc^r^ in both *E. coli* and *B. subtilis*	Purchased from Takara
pHYP43-SPsacB	pHY300PLK derivative carrying P43 promoter, SP *sacB*, *pgdS* gene	This study
pHYP43-SPyvpA	pHY300PLK derivative carrying P43 promoter, SP*yvpA*, *pgdS* gene	This study
pHYP43-SPbprA	pHY300PLK derivative carrying P43 promoter, SP*bprA*, *pgdS* gene	This study
pHYP43-SPaprE	pHY300PLK derivative carrying P43 promoter, SP*aprE*, *pgdS* gene	This study
pHYP43-SPvpr	pHY300PLK derivative carrying P43 promoter, SP*vpr*, *pgdS* gene	This study
pHYP43-SPsacC	pHY300PLK derivative carrying P43 promoter, SP*sacC*, *pgdS* gene	This study
pHYP43-SPggt	pHY300PLK derivative carrying P43 promoter, SP*ggt*, *pgdS* gene	This study
pHYP43-SPpgdS	pHY300PLK derivative carrying P43 promoter, SP*pgdS*, *pgdS* gene	This study
pHYPpgdS-SPbprA	pHY300PLK derivative carrying PpgdS promoter, SP*bprA*, *pgdS* gene	This study
pHYPbprA-SPbprA	pHY300PLK derivative carrying PbprA promoter, SP*bprA*, *pgdS* gene	This study
pHYPbacA-SPbprA	pHY300PLK derivative carrying PbacA promoter, SP*bprA*, *pgdS* gene	This study
pHYPbprA-SPsacB	pHY300PLK derivative carrying PbprA promoter, SP*sacB*, *pgdS* gene	This study
pHYPbprA-SPaprE	pHY300PLK derivative carrying PbprA promoter, SP*aprE*, *pgdS* gene	This study
pHYPbprA-SPsacC	pHY300PLK derivative carrying PbprA promoter, SP*sacC*, *pgdS* gene	This study
pHYPpgdS-SPsacB	pHY300PLK derivative carrying PpgdS promoter, SP*sacB*, *pgdS* gene	This study
pHYPpgdS-SPaprE	pHY300PLK derivative carrying PpgdS promoter, SP*aprE*, *pgdS* gene	This study
pHYPpgdS-SPsacC	pHY300PLK derivative carrying PpgdS promoter, SP*sacC*, *pgdS* gene	This study
pHYPbacA-SPsacB	pHY300PLK derivative carrying PbacA promoter, SP*sacB*, *pgdS* gene	This study
pHYPbacA-SPaprE	pHY300PLK derivative carrying PbacA promoter, SP*aprE*, *pgdS* gene	This study
pHYPbacA-SPsacC	pHY300PLK derivative carrying PbacA promoter, SP*sacC*, *pgdS* gene	This study

*Ori_ts_ thermosensitive replication origin, Kan^r^ kanamycin resistance gene, Amp^r^ ampicillin resistance gene, Tc^r^ tetracycline resistance gene.*

For the seed cultures, *B. licheniformis* cells were precultured in LB medium and incubated at 37°C for 12 h. For γ-PGA fermentation, 250 mL flasks containing 50 mL medium (glucose 80.00 g/L, sodium citrate 30.00 g/L, NH_4_Cl 8.00 g/L, NaNO_3_ 15.00 g/L, K_2_HPO_4_⋅3H_2_O 1.00 g/L, MgSO_4_⋅7H_2_O 1.00 g/L, ZnSO_4_⋅7H_2_O 1.00 g/L, CaCl_2_ 1.00 g/L, MnSO_4_⋅H_2_O 0.15 g/L, pH7.2) were inoculated with 3% (v/v) seed cultures and cultivated at 37°C.

The *B. licheniformis* cells grow in 1-L bioreactor (T&J Bio-engineering Co., Ltd., Shanghai, China) with 0.60 L of media for 10 h. The aeration rate was 1.0 vvm, and the stirring speed was 300 rpm. The volume fractions of exhausted O_2_ and CO_2_ were measured by exhaust gas analyzer, and the volumetric mass transfer coefficient were calculated by the exhaust gas analysis system (T&J Bio−engineering Co., Ltd., Shanghai, China) (Cai et al., 2018).

Batch fermentation was carried out in the 3-L bioreactor (T&J Bio-engineering Co., Ltd., Shanghai, China) containing 1.8 L media with an aeration rate of 1.0 vvm. The agitation speed was starting to set at 300 rpm and increased to 600 rpm after 12 h. Samples were collected periodically to analyze the cell growth, glucose, and γ-PGA concentrations. Δ*pgdS* was further verified by diagnostic PCR and DNA sequencing.

### Construction of Gene Expression Strains

**FIGURE 1:**
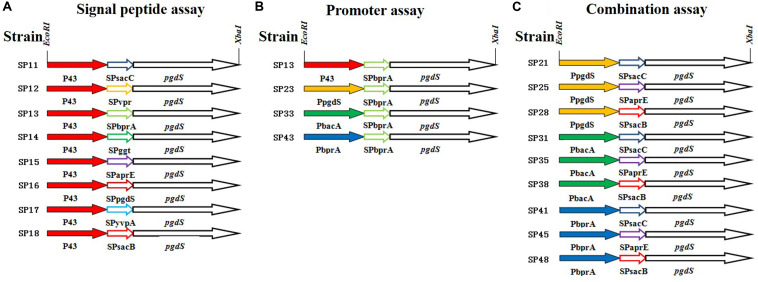
The details of PgdS expression cassettes constructed by manipulating its promoter and signal peptide independently and in combination. **(A)** Signal peptide assay containing SPsacB, SPaprE, SPggt, SPvpr, SPbprA, SPyvpA, SPpgdS, and SPsacC using P43 promoter; **(B)** promoter assay containing P43, PbacA, PpgdS, and PbprA using BprA signal peptide; **(C)** combination assay containing three promoters (PpgdS, PbacA, and PbprA) and three signal peptides (SPsacB, SPpgdS, and SPsacC). Different colors indicate different promoters or signal peptides, and the named rule of strains: first number means promoter, second number means signal peptide.

As an example, the construction procedure of the plasmid pHYP43-SPsacB (containing P43 promoter and SPsacB) was described. Briefly, P43 promoter of *B. subtilis* 168, signal peptide of levansucrase SacB (SPsacB), gene *pgdS* (without its own signal peptide) and *amyL* terminator of *B. licheniformis* WX-02 were amplified and fused by overlapping PCR to obtain the expression cassettes (Figure 1). The expression cassettes were inserted into *Eco*RI*/Xba*I-cut pHY-300PLK, and the resulting plasmids were transformed into WX-02Δ*pgdS* to obtain recombinant strain SP18. Other PgdS expression strains were constructed by the similar method. Notably, all the recombinant vectors were verified by DNA sequencing. Moreover, the empty plasmid pHY300PLK was transformed into WX-02Δ*pgdS* to generate the control strain SP01.

### Enzymatic Assay for PgdS

Crude enzyme solution of PgdS was attained by removing the cells via centrifugation (13 700 × *g*, 20 min). Enzyme activity was measured as described by Ashiuchi et al. (2006). Briefly, the reaction contains 20 μM f potassium phosphate buffer (pH 6.0), 0.2 nM 1.0 × 10^6^ Da γ-PGA substrate, 2 μM dithiothreitol and 10 μL of crude enzyme solution, and was incubated at 37°C for 4 h. After stopped, 100 μL of reaction solution was withdrawn and mixed with 100 μL of 0.2 M borate buffer (pH 8.5) and 20 μL of 10 mmol/L fluorodinitrobenzene (FDNB, in acetone). The hydrolysis of FDNB-modified fragments and the assay of dinitrophenyl glutamate monomers (DNPGlu) were performed as described previously (Ashiuchi et al., 2006). One unit of PgdS was defined as the amount of enzyme that generated 1 nmol of terminal glutamate group per minute.

### SDS-PAGE Analysis

SDS-PAGE analysis was applied to determine the extracellular contents of PgdS in recombinant strains and the BSA (bovine serum albumin) was used as a protein standard. Briefly, 1 mL fermentation broth was centrifuged at 10000 × *g* for 10 min, and the supernatant was precipitated by 6.12 mol/L trichloroacetic acid (TCA). The precipitate was washed with absolute alcohol, and re-dissolved in a 100 uL solution which containing 2 mol/L thiourea and 8 mol/L urea. Protein concentrations were quantitated by Bradford assay (Hammond and Kruger, 1988) and BSA was used as a protein standard. Protein samples as while as BSA solution were mixed with 2 × SDS–PAGE loading buffer containing β-mercaptoethanol in 1:1 ratio, then boiling water bath for 5 min and then samples contain loading buffer were analyzed by the 15% (w/v) gel (Cai et al., 2016). The gels were then imaged with a Bio-Rad’s Gel Doc XR + system (Bio-Rad, United States). The target bands in the gels were visualized and quantified with ImageJ software (Rasband, W.S., ImageJ, United States National Institutes of Health, Bethesda, MD, United States) (Gallagher, 2014), and the BSA proteins were used as standards.

### Quantitative PCR Analysis

When the cells grew into the mid-logarithmic growth phase, the cells were collected for RNA extraction according to Shi’s method (Shi et al., 2019), The HiScript^®^II Q RT SuperMix for qPCR (+gDNA wiper) (Vazyme, China) was employed for cDNA synthesis. The real-time PCR was performed using iTaqTM Universal SYBR^®^ Green Supermix (Bio-Rad, United States). The experiments were performed in three replicates, and *16S rRNA* was used as the reference gene (Rocha et al., 2015). The relative transcriptional level of *pgdS* gene was calculated using the 2^–ΔΔCt^ method.

### Analytical Methods

The cell density (OD_600_) was measured using the 752N spectrophotometer (Shanghai Opler Instrument Co., Ltd., Shanghai, China). The glucose concentration was determined by SBA-40C bioanalyzer (Academy of Sciences, Shandong, China).

The purification of γ –PGA was carried out by the method reported previously (Tian et al., 2014). Number average molecular weight (Mn), weight average molecular weight (Mw), and polydispersity index (Mw/Mn) of γ-PGA were measured using gel permeation chromatography (GPC) with a refractive index (RI) detector and a Shodex OHpak SB-806 HQ column (8.0 mm ID × 300 mm, 13 μm) (Birrer et al., 1994). Pullulan standards of narrow polydispersity (SHANGHAI ZZBIO CO., Ltd., Shanghai, China) were employed to establish a calibration curve. The concentration of γ-PGA was calculated from the peak area of the GPC measurements, with purified γ-PGA as a standard (Birrer et al., 1994; Xu et al., 2019).

### Statistical Analysis

All data are represented as the means of three replicates and bars represent the standard deviations. Data were analyzed by SPSS Statistics software v. 19.0. Pearson correlation coefficient, *t*-test, and ANOVA test were carried out to compare means values and *p* < 0.05 were considered statistically significant.

## Results

To systematically study the influence of PgdS expression level on the Mws of γ-PGAs, the native *pgdS* gene was deleted from the genome of *B. licheniformis* WX-02. PgdS depolymerase was expressed episomally and controlled by the promoter and signal peptide independently and in combination. We employed the strategy to construct twenty recombinant strains with different PgdS hydrolytic activities to generate γ-PGA with different Mws. In our previous study, the nattokinase was used as a reporter to test the effects of signal peptide on protein expression (Cai et al., 2016). According to the data, nattokinase protein with the eight signal peptides (SPsacB, SPaprE, SPpgdS, SPbprA, SPggt, SPsacC, SPyvpA, and SPvpr) expressed in various levels (nearly ten times between the highest level and the lowest level, due to the high copy number of plasmid harboring pgdS gene, we tend to choose the signal peptides with low expression level to avoid over expression of PgdS) (Supplementary Table S2). In order to get multiple expression levels of PgdS, those eight signal peptides and four promoters (P43, PpgdS, PbprA, and PbacA) were employed to construct *pgdS* expression vectors (Figure 1). According to the result of pre-experiment (Supplementary Figure S1A), the PGA titers of each strain were highest at 36 h. The molecular weight of PGA was decreased with the prolonged fermentation (Supplementary Figure S1B), while, at the later period of fermentation (30–42 h), the γ-PGA molecular weight values for the recombinants SP12, SP14, and SP18 were maintained. So the time of fermentation of all the strains were set as 36 h.

### Modulating PgdS Secretion to Tune γ-PGA Molecular Weight

All PgdS expression vectors were transferred into the *pgdS* deletion strain WX-02Δ*pgdS*, and the resultant strains were designated *B. licheniformis* SP11–SP18, respectively (Table 1).

**FIGURE 2:**
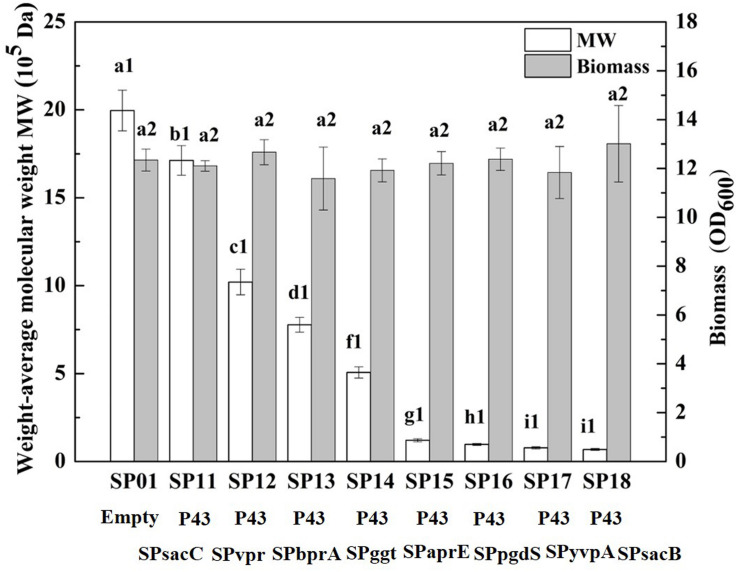
Comparison of Mws and biomass in *B. licheniformis* under different signal peptides. The data are presented as the mean ± SD of three replications. Different lowercase letters indicate significant differences among strains at 0.05 level (*p* < 0.05).

In our previous study, we have construct SP library in *B. licheniformis* using nattokinase as reporter (Cai et al., 2016). We choose eight different SP from *B. licheniformis* to expression PgdS. The SDS-PAGE analysis of the culture supernatants (Supplementary Figure S2) showed a band of the predicted molecular mass (4.2 × 10^4^ Da) among the SP12–SP18 strains, except the control strain SP01 and SP11 (carrying cassette with the combination of P43-SPsacC). Among these strains, the secretion efficiency ratios were SPsacB > SPyvpA > SPpgdS > SPaprE > SPggt > SPbprA > SPvpr > SPsacC (Table 2). As indicated in Figure 2, overexpression of PgdS enabled efficient reduction of Mws, which was consistent with the results reported by Tian et al. (2014) and Sha et al. (2018). The PgdS overexpression strains also produced γ-PGA with lower Mns values and polydispersity index (Table 2). The Mws of γ-PGAs produced by recombinant strains were ranged from (6.82 ± 0.51) × 10^4^ to (1.71 ± 0.08) × 10^6^ Da, compared with the control strain (1.99 ± 0.11) × 10^6^ Da. Interestingly, cell growth showed no significant differences between the recombinant strains and control strain SP01. Therefore, our results indicated that γ-PGA with a wide range of Mws (6.82 × 10^4^–1.71 × 10^6^ Da) could be achieved via modulating the secretory capability of PgdS.

**TABLE 2:** The γ-PGA yields of recombinant strains and the PgdS concentrations of culture supernatants.

Strain	Mw (×10^5^ Da)	Mn (×10^5^ Da)	Polydispersity index	γ-PGA yields (g/L)	PgdS protein concentrations of culture supernatant (mg/L)	Total protein concentrations of culture supernatant (mg/L)	PgdS activity (U/mL)
SP01	19.96 ± 1.16	13.49 ± 0.89	1.48 ± 0.07	25.65 ± 0.56	ND	83.4 ± 4.2	0
SP41	19.10 ± 0.75	13.08 ± 0.78	1.46 ± 0.04	26.86 ± 0.94	2.4	112.4 ± 11.3	0.72 ± 0.23
SP43	17.80 ± 0.87	12.11 ± 0.86	1.47 ± 0.06	27.36 ± 0.73	ND	103.7 ± 17.3	0.54 ± 0.33
SP31	17.43 ± 0.56	12.10 ± 0.54	1.44 ± 0.03	27.88 ± 0.61	3.3	115.1 ± 12.3	1.25 ± 0.21
SP21	17.40 ± 0.11	12.03 ± 0.77	1.45 ± 0.03	27.14 ± 1.14	4.2	127.3 ± 17.8	1.22 ± 0.17
SP11	17.13 ± 0.84	12.06 ± 0.61	1.42 ± 0.05	27.09 ± 0.99	ND	121.9 ± 11.5	1.09 ± 0.37
SP45	16.72 ± 0.88	11.77 ± 0.92	1.42 ± 0.06	27.87 ± 0.62	7.7	121.3 ± 7.6	6.37 ± 0.42
SP33	14.41 ± 0.67	10.44 ± 1.02	1.38 ± 0.07	28.19 ± 0.48	7.7	112.7 ± 9.5	6.71 ± 0.21
SP48	13.40 ± 0.73	9.64 ± 0.55	1.39 ± 0.04	28.75 ± 0.38	8.6	128.8 ± 14.7	7.22 ± 0.33
SP12	10.21 ± 0.73	7.51 ± 0.43	1.36 ± 0.05	28.32 ± 0.56	9.1	115.1 ± 7.2	8.17 ± 0.54
SP23	9.55 ± 0.46	7.07 ± 0.50	1.35 ± 0.01	28.68 ± 0.71	9.9	124.1 ± 8.1	8.68 ± 0.38
SP13	7.78 ± 0.42	5.72 ± 0.67	1.36 ± 0.03	29.27 ± 1.28	12.5	127.5 ± 17.9	10.05 ± 0.21
SP14	5.06 ± 0.32	3.80 ± 0.31	1.33 ± 0.01	31.58 ± 1.49	13.3	122.3 ± 15.5	12.12 ± 0.26
SP35	3.19 ± 0.16	2.40 ± 0.27	1.33 ± 0.03	32.67 ± 0.59	14.5	128.0 ± 16.2	13.22 ± 0.38
SP15	1.21 ± 0.08	0.92 ± 0.13	1.31 ± 0.01	31.45 ± 1.39	25.1	124.3 ± 10.3	22.18 ± 0.51
SP25	1.18 ± 0.10	0.90 ± 0.15	1.31 ± 0.01	33.30 ± 1.06	28.6	146.6 ± 15.0	25.26 ± 0.40
SP38	1.12 ± 0.10	0.85 ± 0.07	1.32 ± 0.02	33.80 ± 0.97	28.4	143.2 ± 18.5	25.13 ± 0.37
SP16	0.97 ± 0.05	0.76 ± 0.07	1.27 ± 0.01	34.00 ± 0.12	40.7	165.6 ± 14.7	26.18 ± 0.62
SP17	0.78 ± 0.06	0.63 ± 0.05	1.24 ± 0.01	34.46 ± 0.75	41.4	161.8 ± 11.4	26.38 ± 0.49
SP28	0.77 ± 0.08	0.62 ± 0.06	1.25 ± 0.01	34.33 ± 1.21	40.9	155.6 ± 18.1	26.12 ± 0.45
SP18	0.68 ± 0.05	0.56 ± 0.05	1.22 ± 0.01	34.60 ± 0.65	42.7	158.9 ± 16.6	32.22 ± 0.39

*The assays of molecular weights, γ-PGA yields were performed in triplicate. The total protein concentrations of fermentation supernatant were converting from the concentrations of re-dissolved precipitate and the errors are shown as standard deviations. Mw emphasizes the influence of the part with the highest molecular weight to the average molecular weight, while Mn emphasizes the influence of the part with the largest molecular number in a certain range to the average molecular weight. The value of polydispersity index (Mw/Mn) reflects the uniformity of particle size, the lower the value, the more uniform of the grain size.*

### Modification of γ-PGA Molecular Weight by Regulating *pgdS* Transcriptional Level

According to the above results, the Mws of γ-PGAs could be tuned by PgdS secretion. We then evaluated the effect of the transcriptional levels of *pgdS* on Mws by using promoters with different strength. Four promoters P43, PpgdS, PbacA, and PbprA were applied to drive the transcription of *pgdS*. The four recombinant vectors harboring the signal peptide BprA were constructed and transformed into the WX-02Δ*pgdS*, generated four recombinant strains SP13, SP23, SP33, and SP43, respectively (Table 1).

**FIGURE 3:**
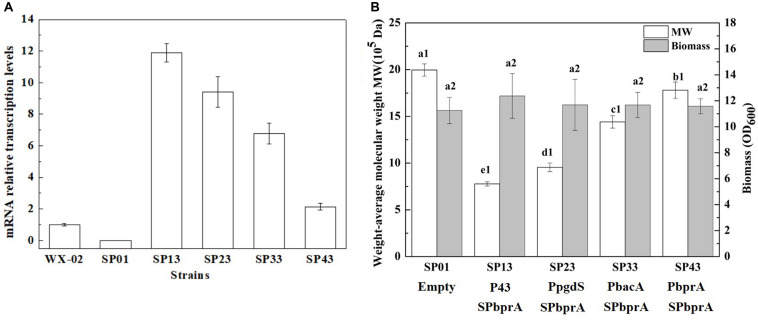
The transcriptional levels of gene *pgdS* among strains with different promoters **(A)**. Data are expressed as mean ± SD of three replications. Comparison of Mws and biomass in *B. licheniformis* under different promoters **(B)**. The data are presented as the mean ± SD of three replications. Different lowercase letters indicate significant differences among strains at 0.05 level (*p* < 0.05).

To examine the expression efficiency of the four promoters, the transcriptional levels of *pgdS* driven by these promoters were determined by real-time PCR. The results showed that the transcription levels from P43, PpgdS, and PbacA were increased in different degrees compared with the PbprA, and P43 exhibited the highest transcription level (*p* < 0.05) (Figure 3A). The expression strength of different promoters was also determined by SDS-PAGE assay (Supplementary Figure S3), and the concentrations of PgdS were quantified (Table 2), the results confirmed that the expression strength of these four promoters was in a descending order of P43 > PpgdS > PbacA > PbprA.

After 36 h cultivation, we measured the Mws and yields of γ-PGA produced by these recombinant strains. The Mws of the polymer produced by SP13, SP23, SP33, and SP43 were (7.78 ± 0.23) × 10^5^, (9.55 ± 0.46) × 10^5^, (14.41 ± 0.67) × 10^5^, and (17.80 ± 0.87) × 10^5^ Da, respectively (Figure 3B), which decreased by 61.02, 52.15, 27.81, and 10.82%, respectively, compared to the control strain SP01 (19.96 × 10^5^ Da). However, the cell growth of these four recombinant strains were approximately the same as that of SP01. Collectively, our results showed that an apparent reduction of Mws along with the enhanced promoter strength.

### Varying Levels of PgdS Expression Enable Controlling the Molecular Weights of γ-PGAs

In order to further boost the performance, the promoter and signal peptide were combined to regulate PgdS expression. Three promoters [PpgdS(H), PbacA(M) and PbprA(L)] and signal peptides [SPsacB(H), SPpgdS(M), SPsacC(L)] were selected and combined to form nine expression cassettes (Figure 1). Every expression cassette was cloned in pHY300PLK, and the resultant vectors were transformed into WX-02Δ*pgdS*. The transformed strains were designated as *B. licheniformis* SP21, SP25, SP28, SP31, SP35, SP38, SP41, SP45, and SP48, respectively.

**FIGURE 4:**
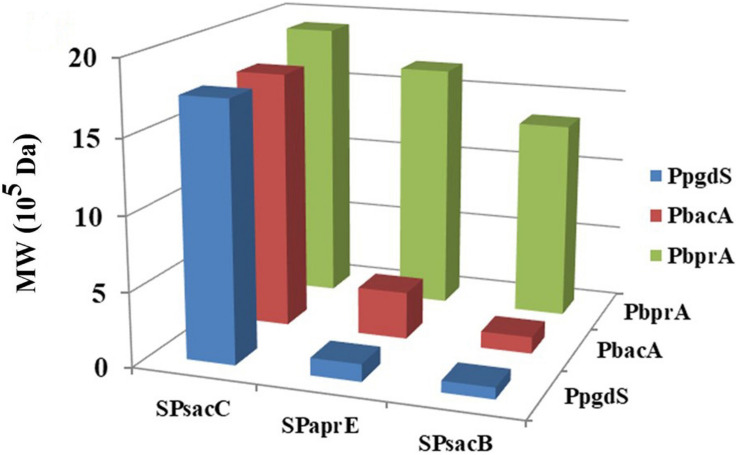
The Mws of γ-PGA in recombinant strains (SP01, SP41, SP45, SP48, SP31, SP35, SP38, SP21, SP25, and SP28). Data are expressed as mean ± SD of three replications, *M*: protein marker.

As shown in Table 2, the activities of PgdS in strains SP28, SP25, and SP38 (26.12 ± 0.45, 25.26 ± 0.4, and 25.13 ± 0.37 U/mL) were significantly greater among all strains, and SP35 (13.22 ± 0.38 U/mL) and SP48 (7.22 ± 0.33 U/mL) exhibited moderate activity. In contrast, weak enzyme activities were detected in strains SP21, SP31, SP41, and SP45. The results of SDS-PAGE assay (Supplementary Figures S4, S5) and the concentrations of PgdS protein of each strains (Table 2) were consistent with PgdS activity assay. The Mws of γ-PGA produced by these combinations were measured at 36 h (Figure 4). The Mws of γ-PGA mediated by the nine combinations varied greatly from (7.74 ± 0.80) × 10^4^ to (1.91 ± 0.08) × 10^6^ Da. To assess the relationship between PgdS expression and γ-PGA Mws, the Pearson correlation coefficients were calculated (Weaver and Wuensch, 2013). A correlation coefficient of −0.945 indicated a strong inverse relation between extracellular PgdS activity and γ-PGA Mws (*P* < 0.01) (Supplementary Figure S6A). Taken together, engineering PgdS expression cassette was an efficient approach to produce γ-PGAs with specific Mws.

### Increasing γ-PGA Yield Along With Reduced Molecular Weights

In this study, we achieved the production of low-, medium-, and high-Mw γ-PGA in the same microbial chassis. The effect of PgdS expression levels on γ-PGA yield was further studied (Table 2). We found that the γ-PGA yield in recombinant strains was obviously increased compared to the control strain when the Mws of γ-PGA decreased by over 50%. A dispersion diagram was obtained with the PgdS yields and logarithms of γ-PGA molecular weight (Supplementary Figure S6B), showing the increasing γ-PGA yield along with the reduction of molecular weights (Pearson correlation coefficient = −0.958). Furthermore, the strain SP18 produced 34.60 g/L γ-PGAs, which increased by 34.89% compared with the control strain (25.65 g/L).

**FIGURE 5:**
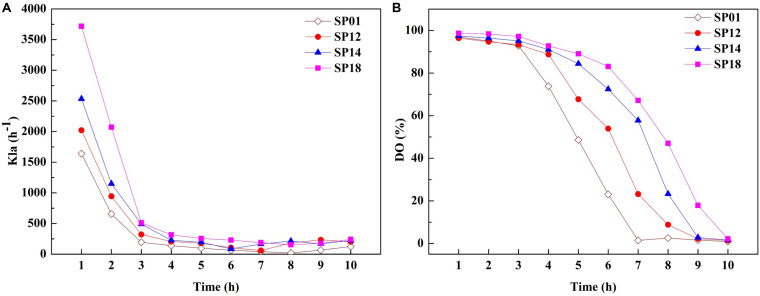
Comparison of *k*_L_a **(A)** and dissolved oxygen (DO) **(B)** between control strain SP01 and recombinant strains in a 1-L fermenter. Time courses of recombinant strains SP01, SP12, SP14, and SP18 in batch culture. The *k*_L_a and DO were measured and calculated at regular intervals.

The viscosity of fermentation broth affects the oxygen transfer rate (OTR), which is proportional to the volumetric mass transfer coefficient and substrate utilization. The OTR plays vital roles in the growth of strains and the production of target metabolites (Damiani et al., 2014). We hypothesized that improvement of γ-PGA yield in engineered strains may be due to the higher OTR. To prove this hypothesis, the OTRs of fermentation broths from the *B. licheniformis* SP01 (Mw, 1.99 × 10^6^ Da), SP12 (Mw, 1.02 × 10^6^ Da), SP14 (Mw, 5.06 × 10^5^ Da), and SP18 (Mw, 6.82 × 10^4^ Da) were compared in 1-L fermenter (Figure 5). All recombinant strains showed improved *k*_L_a compared to the control strain SP01 throughout the fermentation process. Among them, *k*_L_a and DO were consistent with the trend of values negatively correlated with the γ-PGA Mws in these recombinant strains (Figures 5A,B). These results confirmed that low viscosity of fermentation broth could improve γ-PGA yield by increasing the oxygen transfer rate.

### Large-Scale Fermentation of Recombinant *B. licheniformis* in 3-L Fermenter

**FIGURE 6:**
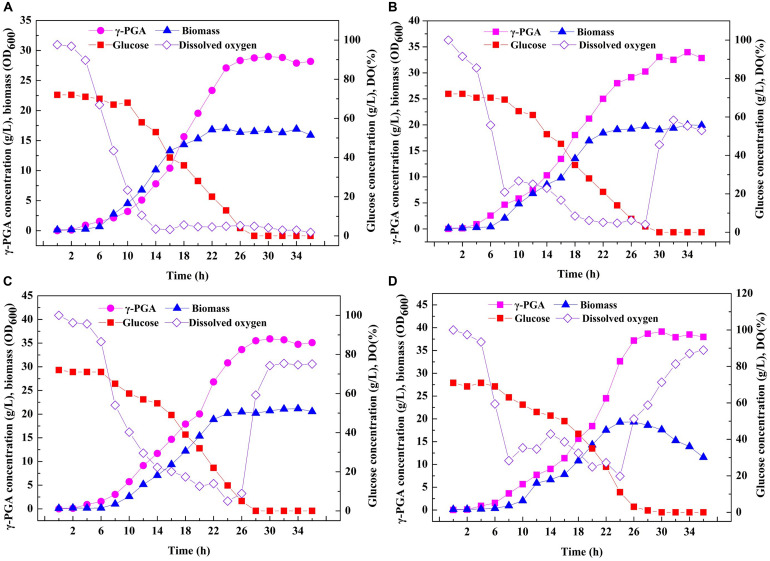
Large-scale fermentation of recombinant *B. licheniformis* strains in 3-L fermenter. Time courses of recombinant strains SP01 **(A)**, SP12 **(B)**, SP14 **(C)**, and SP18 **(D)** in batch culture. γ-PGA concentration, biomass (OD_600_), glucose concentration and DO were measured and calculated at regular intervals.

Based on the above results, an efficient system for one-step synthesis of γ-PGA with a wide range of Mws (6.82 × 10^4^–1.99 × 10^6^ Da) was developed by regulating PgdS expression. Therefore, in order to further explored the applicability of this system in large-scale γ-PGA production, the engineered strains SP01, SP12, SP14, and SP18 were carried out in a 3-L bioreactor, respectively (Figure 6). Compared to the control strain SP01, the DO values in cultures of SP12, SP14, and SP18 were maintained at higher levels, resulting in higher cell growth rates and biomass (Figure 6). The highest yield of γ-PGAs produced by the SP01 was 29.00 g/L at 30 h (Figure 5A). In contrast, the maximal γ-PGA yields in strains SP12, SP14, and SP18 reached 33.05, 35.91, and 39.13 g/L, respectively, increasing by 12.25, 23.83, and 34.93% (Figures 5B–D), and the Mws of γ-PGAs in corresponding strains were 1.42 × 10^6^, 5.56 × 10^5^, and 7.83 × 10^4^ Da, respectively. Therefore, our results demonstrated that it was feasible and efficient to produce specific γ-PGAs using glutamate-free medium by the controllable expression of PgdS.

## Discussion

Since γ-PGAs with different Mws show specific applications, the Mws must be precisely controlled. γ-PGAs produced by a single strain was often difficult to meet the requirements of molecular weight differentiation (Supplementary Table S4). In this work, we aimed to develop a convenient system for one-step production of regulated-molecular-weight γ-PGAs through the controlled expression of PgdS depolymerase by manipulating the promoters and signal peptides independently and in combination.

Currently, many endo-type γ-PGA hydrolases (PgdS) has been characterized from various strains (Suzuki and Tahara, 2003; Tian et al., 2014) and used as a target to improve the yields of γ-PGAs (Scoffone et al., 2013; Sha et al., 2018). It has been proved that PgdS degraded γ-PGA in the extracellular region (Yao et al., 2009). The optimization of signal peptides is an efficient method to increase the secretion of desirable proteins (Zhang et al., 2016). According to Cai et al., 81 signal peptides were identified in *B. licheniformis* WX-02 by the SignalP tool^1^ (Cai et al., 2016). The effects of all signal peptides on the secretion of nattokinase from *B. subtilis* MBS 04-6 were investigated, and the strains with different signal peptides showed different activities ranging from 0 to 31.99 FU/mL (Cai et al., 2016). In this study, we investigated the effects of eight proteases signal peptides (SacC, Vpr, BprA, GGT, AprE, PgdS, YvpA, and SacB) on PgdS secretion and γ-PGA Mws. Our results showed that higher γ-PGA degradation capability was achieved with the increased PgdS secretion, which was consistent with the previous reports (Yuan et al., 2015; Jin et al., 2016). Besides, the optimal signal peptides varied for different proteins (Degering et al., 2010). In this study, the maximum activity of PgdS being obtained under the signal peptide of SPsacB in *B. licheniformis* among the selected signal peptides (Table 2), which is different with the optimal signal peptide (SPaprE) for nattokinase secretion (Cai et al., 2016).

The promoter is another critical element for controlling the protein expression (Wang et al., 2014). There are many classes of promoters that could be employed to dynamically regulate the expression of protein, such as inducible promoters, constitutive promoters of different strengths, condition-responsive promoters, and growth phase responsive promoters (Fontana et al., 2018; Maury et al., 2018; Tekel et al., 2019). In this work, we explore the effects of *pgdS* transcriptional levels on γ-PGA Mws using promoters with different strengths (P43, PbacA, PbprA, and PpgdS). P43 is considered as a strong promoter for protein expression (Zhang et al., 2007). PpgdS is the native promoter of PgdS depolymerase and regulated by sigma D factor from *B. licheniformis* (Mitsunaga et al., 2016). PbacA is the promoter of *bacABC* operon involved in bacitracin synthesis from *B. licheniformis* DW2 (Shu et al., 2018). PbprA, the promoter of bacillopeptidase F (BprA), is a weak promoter according to our transcriptome data (data not shown) and used as a control. Among the four promoters, P43 promoter is the most effective one to drive the expression of *pgdS*.

In order to further evaluate the effect of regulated PgdS expression on the Mws of γ-PGAs, we explored various levels of PgdS expression by combining promoters and signal peptides. Through this strategy, the combined strains provided γ-PGA with a wide range of Mws [(7.74 ± 0.80) × 10^4^–(1.91 ± 0.08) × 10^6^ Da]. Based on these results, regulating PgdS expression was an efficient and simple method to obtain γ-PGAs with specific Mws. Compared to other methods, we could achieve broader range of Mws of γ-PGAs, which is beneficial to broaden the industrial application of γ-PGAs. Previous studies have mainly focused on increasing the production of high-Mw γ-PGAs (Birrer et al., 1994; Scoffone et al., 2013), limiting the applications of γ-PGAs in other fields. However, one limitation of this study is that the determination of the correlations between the PgdS expression and γ-PGA Mws is based on small dataset (*n* = 9). We will achieve precise control of Mws via the titratable regulation of PgdS expression using the further development of tools.

Specially, all recombinant strains containing *pgdS* gene produced higher γ-PGA yields compared with the control strain SP01, which confirmed that overexpression of PgdS is an efficient approach to enhance γ-PGA production. Due to the high viscosity of high-Mw γ-PGAs, it severely decreases oxygen transfer in the fermentation process, resulting in inhibition of cell growth and limit of γ-PGA production (Su et al., 2010). In this paper, the k*_L_*a and DO of fermentation broths in recombinant strains were improved compared to the control strain SP01 with high-Mw γ-PGA. One possible explanation for the increased DO was that the reduced Mws decreased the viscosity of the fermentation broth, enabling higher oxygen transfer rate, further improving cell growth, substrate utilization and γ-PGA yields.

Ultimately, we investigated the applicability of the system in the large-scale production of specific-molecular-weight γ-PGAs. Our results indicated that the γ-PGA could be efficiently produced on a high level (29.00–39.13 g/L) without additional glutamic acid using the recombinant strains in 3-L fermenter. The results from this work and related studies were summarized in Table 3. In particular, the highest yield of γ-PGA (39.13 g/L) with molecular-weight value of 7.83 × 10^4^ Da was obtained in SP18 strain, which was 34.93% higher than that of control strain SP01, and the yield was higher than those produced by most γ-PGA producers (Table 3). Moreover, the γ-PGA productivity was 1.30 g/L/h, which was the highest from glutamate-free medium to date. One possible explanation for the increased DO was that the reduced Mws decreased the viscosity of the fermentation broth, enabling higher oxygen transfer rate, further improving cell growth, substrate utilization and γ-PGA yields. Collectively, it is more applicable to industrially produce specific-molecular-weight γ-PGAs using our strategies than previously reported studies.

**TABLE 3:** Comparison of γ-PGA production from glutamic acid independent stains.

Strains	Key nutrients (g/L)	Yield g/g	Titer (g/L)	Productivity (g/L/h)	References
*B. subtilis* C1	Glycerol, citric acid, NH_4_Cl	144	21.4	0.15	Shih et al., 2005
*B. licheniformis* A13	Glucose, NH_4_Cl, yeast extract	72	28.2	0.39	Mabrouk et al., 2012
*B. subtilis* C10	Glucose, NH_4_Cl	32	27.7	0.87	Zhang et al., 2012
*B. licheniformis* TISTR 1010	Glucose, citric acid, NH_4_Cl	77	27.5	0.29	Kongklom et al., 2015
*B. licheniformis* TISTR 1010	Glucose, citric acid, NH_4_Cl	43	39.9	0.93	Kongklom et al., 2017
*B. amyloliquefaciens* NB (pNX01-*pgdS*1)	Raw inulin extract, glutamate, (NH_4_)_2_SO_4_	72	17.62		Sha et al., 2018
*B. licheniformis* BC4	Glycerol, sodium citrate, NaNO_3_, NH_4_Cl	48	19.20	0.40	Zhan et al., 2018
*B. licheniformis* SP18	Glucose, sodium citrate, NaNO_3_, NH_4_Cl	30	39.13	1.30	This study

In conclusion, we developed an efficient system for one-step production of specific Mws γ-PGA through regulating the PgdS expression in *B. licheniformis* for the first time. The ability to produce γ-PGA with low, medium, and high Mws ranging between 6.82 × 10^4^ Da and 1.99 × 10^6^ Da was demonstrated by manipulating the promoter and signal peptide independently and in combination. The maximum production of γ-PGA (Mw, 7.83 × 10^4^ Da) reached 39.13 g/L from glutamate-free medium in batch fermentation. Our study presented a potential method for commercial production of specific-molecular-weight γ-PGA, and this strategy could also be used to produce other biopolymers by precisely controlling corresponding depolymerase expression.

## Data Availability Statement

All datasets generated for this study are included in the article/Supplementary Material.

## Author Contributions

SC and FS designed and supervised the study. HW, DW, YX, JD, and JC performed the experiments. DW and HW analyzed the data and wrote the manuscript. YZ, QW, DC, SC, and FS revised the manuscript. All authors read and approved the final manuscript.

## Conflict of Interest

The authors declare that the research was conducted in the absence of any commercial or financial relationships that could be construed as a potential conflict of interest.

## Footnotes

1 http://www.cbs.dtu.dk/services/SignalP/
