# Supplementary material for: Engineering Expression Cassette of pgdS for Efficient Production of Poly-γ-Glutamic Acids With Specific Molecular Weights in Bacillus licheniformis
Source: Front Bioeng Biotechnol. 2020 Jul 9;8:728. doi: 10.3389/fbioe.2020.00728 (PMC7381323; doi:10.3389/fbioe.2020.00728)
Supplement: Supplementary file 1 [file Data_Sheet_1.docx]

**Table S1 Primers used for PCR in this study**

| **Primer name** | **Sequence of primer (5' to 3')^a^** |
| --- | --- |
| pHY-F | CAGATTTCGTGATGCTTGTC |
| pHY-R | GTTTATTATCCATACCCTTAC |
| P43-F(*Eco*RI) | CG**GAATTC**TGATAGGTGGTATGTTTTCG |
| P43-R | TTCATGTGTACATTCCTCTC |
| *cwlO*-F | GAGAGGAATGTACACATGAAGTGAAGAAAAAGGTTTATAC |
| *cwlO*-R | AATCCGTCCTCTCTGCTCTTTTATTCAACGACCCGTCTTAC |
| *ggt*-F | GAGAGGAATGTACACATGAAATGAGACGGTTAGCTTTCT |
| *ggt*-R | AATCCGTCCTCTCTGCTCTTCTATTTAGCCGATGTCTT |
| TamyL-F | AAGAGCAGAGAGGACGGATT |
| TamyL-R(*Xba*I) | GC**TCTAGA**CGCAATAATGCCGTCGCACTG |
| T2-F | ATGTGATAACTCGGCGTA |
| T2-R | GCAAGCAGCAGATTACGC |
| *cwlO*-AF(*Xba*I) | GC**TCTAGA**TCGGCAAACCAATACCTT |
| *cwlO*-AR | TGTTGTCGCACCGAATCCGATTTCTTTCCGCTTGCT |
| *cwlO*-BF | AGCAAGCGGAAAGAAATCGGATTCGGTGCGACAACA |
| *cwlO*-BR(*Sac*I) | C**GAGCTC**TGATGCTGGGCGGTGATT |
| *cwlO*-YF | AAGGCACGGAATCAGTCA |
| *cwlO*-YR | TTTGGCAAAGCGGGTGGA |
| *ggt*-AF(*Xba*I) | GC**TCTAGA**AAGGGTTGTCTGATTCGT |
| *ggt*-AR | GGCTCTTCAATCGCATCCTCGGCTCTGTTACATTCA |
| *ggt*-BF | TGAATGTAACAGAGCCGAGGATGCGATTGAAGAGCC |
| *ggt*-BR(*Sac*I) | C**GAGCTC**AACCGACCAGACCCAATA |
| *ggt-*YF | ATTTTCCGCCAGCAATTC |
| *ggt-*YR | GACATGAAATAAAGCGAG |
| *pgdS*-AF(*Xba*I) | GC**TCTAGA**AAAGGAACAAACCTCGACTGGA |
| *pgdS*-AR | CGAACGATCTGTCAATGTTTTGCGGTTCCCATCCCGAATTG |
| *pgdS*-BF | CAATTCGGGATGGGAACCGCAAAACATTGACAGATCGTTCG |
| *pgdS*-BR(*Sac*I) | C**GAGCTC**CGGGGGAACGGTCCACGA |
| *pgdS*-YF | TTTCTACAGCCTCGGCAACT |
| *pgdS*-YR | CGGCAAACTGCTCTTACA |
| SPsacB-F | GAGAGGAATGTACACATGAAATGAACATCAAAAACATTGCTAA |
| SPsacB-R | CTATTTTCTCGCCGATCGTATCTGCAAAGGTTTGCGGCGCA |
| SPyvpA-F | GAGAGGAATGTACACATGAAATGAAGAGATTAGCAGGT |
| SPyvpA-R | CTATTTTCTCGCCGATCGTATCGGCCAAAGCTTTTTCAGG |
| SPbprA-F | GAGAGGAATGTACACATGAAGTGAAGAAAAAGCCATTATT |
| SPbprA-R | CTATTTTCTCGCCGATCGTATCAGCCTGCACAGCAGCCGGA |
| SPaprE-F | GAGAGGAATGTACACATGAAATGATGAGGAAAAAGAGTT |
|  |  |
| **Table S1 (continued)** | |
| **Primer name** | **Sequence of primer (5' to 3')^a^** |
| SPaprE-R | CTATTTTCTCGCCGATCGTATCAGCAGAAGCGGAATCGCTGAAT |
| SPpgdS-F | GAGAGGAATGTACACATGAATTGATAAAAAAAGCGGCAAA |
| SPpgdS-R | CTATTTTCTCGCCGATCGTATCGGCGCGTACATCATTATGAT |
| SPvpr-F | GAGAGGAATGTACACATGAATTGAGAAAAAGTATCGTGCG |
| SPvpr-R | CTATTTTCTCGCCGATCGTATCTGCCTGCACTCCGGTGAGGAAT |
| SPggt-F | GAGAGGAATGTACACATGAAATGAGACGGTTAGCTTTC |
| SPggt-R | CTATTTTCTCGCCGATCGTATCTTTCGACACCGGACTGAA |
| SPsacC-F | GAGAGGAATGTACACATGAAATGAAAAAGAGAATGATT |
| SPsacC-R | CTATTTTCTCGCCGATCGTATCAGCAGCTGCGGAAAAGGC |
| pgdS-F | GATACGATCGGCGAGAAAATAG |
| pgdS-R(*Xba*I) | GC**TCTAGA**CTACTCAATTCTGACGCTTCC |
| PpgdS-F(*Eco*RI) | CG**GAATTC**TGAAAGGGAAACACGACA |
| PpgdS-R | TTGATCTGTTCCTCCTCTTC |
| SPbprA-F | TGAAAGGGAAACACGACAGTGAAGAAAAAGCCATTATT |
| PbprA-F(*Eco*RI) | CG**GAATTC**CAATGAAAACTTAAAAGACG |
| PbprA-R | ATGACATCCTCCTTTAAGCA |
| SPbprA-F | CAATGAAAACTTAAAAGACGGTGAAGAAAAAGCCATTATT |
| PbacA-F(*Eco*RI) | CG**GAATTC**CCTGCGATTTCGGCGAGATT |
| PbacA-R | ATAAAAATTCTCCTTTTTGAT |
| SPbprA-F | CCTGCGATTTCGGCGAGATTGTGAAGAAAAAGCCATTATT |
| PbprA/SPsacB-F | TGCTTAAAGGAGGATGTCATATGAACATCAAAAACATTGCTAA |
| PbprA/SPaprE-F | TGCTTAAAGGAGGATGTCATATGATGAGGAAAAAGAGTT |
| PbprA/SPsacC-F | TGCTTAAAGGAGGATGTCATATGAAAAAGAGAATGATT |
| PpgdS/SPsacB-F | GAAGAGGAGGAACAGATCAAATGAACATCAAAAACATTGCTAA |
| PpgdS/SPaprE-F | GAAGAGGAGGAACAGATCAAATGATGAGGAAAAAGAGTT |
| PpgdS/SPsacC-F | GAAGAGGAGGAACAGATCAAATGAAAAAGAGAATGATT |
| PbacA/SPsacB-F | ATCAAAAAGGAGAATTTTTATATGAACATCAAAAACATTGCTAA |
| PbacA/SPaprE-F | ATCAAAAAGGAGAATTTTTATATGATGAGGAAAAAGAGTT |
| PbacA/SPsacC-F | ATCAAAAAGGAGAATTTTTATATGAAAAAGAGAATGATT |

^a^The underlines indicates an overlap region for splicing overlap extension PCR (SOE-PCR); Generated restriction site in bold.

Table S2 Signal peptides and corresponding nattokinase yields.

| Signal peptides | Amino acid sequences of signal peptides | nattokinase yields (FU/mL) |
| --- | --- | --- |
| AprE | MMRKKSFWLGMLTAFMLVFTMAFSDSASA | 31.99$\pm$1.55 |
| BprA | VKKKPLFSTFMCAALIGSLLAPAAVQA | 22.68$\pm$2.86 |
| Vpr | LRKSIVRYFVMAFILLFALSTFLTGVQA | 8.72$\pm$0.35 |
| SacB | MNIKNIAKKASALTVAAALLAGGAPQTFA | 6.57$\pm$2.07 |
| YvpA | MMKRLAGTVILSGLLVCGFGQALPEKALA | 5.73$\pm$0.53 |
| SacC | MKKRMIQMGIIGAMMFPEAFSA | 4.16$\pm$0.53 |
| Ggt | MRRLAFLVVAFCLAVGCFFSPVSKA | 3.17$\pm$1.14 |
| PgdS | MIKKAANKKLVLFCGIAVLWMSLFLTNHNDVRA | No detect |

Table S3 The promoters used in this study

| Promoter | DNA sequence |
| --- | --- |
| P43 | TGATAGGTGGTATGTTTTCGCTTGAACTTTTAAATACAGCCATTGAACATACGGTTGATTTAATAACTGACAAACATCACCCTCTTGCTAAAGCGGCCAAGGACGCTGCCGCCGGGGCTGTTTGCGTTTTTACCGTGATTTCGTGTATCATTGGTTTACTTATTTTTTTGCCAAAGCTGTAATGGCTGAAAATTCTTACATTTATTTTACATTTTTAGAAATGGGCGTGAAAAAAAGCGCGCGATTATGTAAAATATAAAGTGATAGCGGTACCATTATAGGTAA  GAGAGGAATGTACACATGAA |
| PpgdS | TGAAAGGGAAACACGACAATTTTCTTAACCGATCAGTGTATAAAGTTTTATAGAAAATCAGGAGGATATATACATGGTTTTGGGGTTCATGTTTATTGTATTCTTTTGAAGGGAATAAAAACTGACAAATTTCGACTGAAGCAAAATTTGAAAATGCATCACCTTACCAATTCGGGATGGGAACCGCACCTCATGTTCATGACCTCTTTAGAATATTTCCCTTCATCTTTTTAATCCGCGCTTAGGTGAAAAAGCTGATCATGCTGTGCTGAGCGTTTCTTCTCGCTATGACGCTGCTGTACATGCAAAAAAAGTCCTTTAAATATCCCAGTTGAATGACGATGAAAGAGGAAAGAAGAGGAGGAACAGATCAA |
| PbacA | CCTGCGATTTCGGCGAGATTCAAGCCCGGGTCTAATCTATTTTTCCTTCTTCGGACGCTTCAAAAATTACTTTTATTATAATCGGAACAGTGTTTTTTAGATCTTTTGATCTATTTGGTGTTTATCTTGTCTCATAAATACATGTTTAAACAATGTAAAATATAAAATATCCAATTCATAAAAAATTAACCATTATTAAACAATATTCCTATGGAAAATAATGATTATTTTTGATAATCTGTTTTCACAAGACGGAGGTTCAATAAAAAATCGGTAAAAGAGCAACTACAGACCAATATTATGGTGAATATTTTATCAAAAAGGAGAATTTTTAT |
| PbprA | CAATGAAAACTTAAAAGACGAGATCGTCGTTACGGTGATCGCGACTGGATTTATCGAACAGGATCAAGATTCATCAAAGCCGCAGAGACCTTTAAACCAAGGCTTAAAACAGCATCATCAGCCTGCTCCGAAACGGGAGCCGAAGCGCGAGGAGCCAAGCATGCCGCATCGCAGCCCATCACAGCCTGCCGAGGATACGCTTGACATCCCGACTTTCTTAAGAAACCGCAATAAACGCTAAACGTCTACAATACATTGGAAAGCTGCCGACAAGAAGTCGGCGGCTTTTTTTGCGCGCTGGAACAGCAGCCGATGTTCCTTCCCCTCCAAAAGCATCAATTGTCTTCTTGATGGAAGCGGCTCCCTCAAAAGCCTTGCATCTAATCTCTCTCTTTTCTCCCGAATGTACAGATTTTCCTTTTTTCTTGTCATCAGGCCGAACTATGAACGGTGAACAAGTCATATGTAAAAAGAACGAATTTGCAAACTGACAAAGGAAAAATGACAGAATACGACAATATCTATAAACCTTTTCTATTACCATTATTAATAGATTCTGTTTCTAGACATAGAAACGCAAGAAACGACTATATTTGCTATTGCTTAAAGGAGGATGTCAT |
| Pxyl | TTAAAATCTCTCGTTCATAAACCGTTCCAGAAAACGTTCGGTTACAAGGGAGGACATCCCAAGCGCTGAGGCGGTGCGGCCGAGCGTGGAGATTTTTAAATGATAATTAGACAAGGCTTCTGCTGCGCTTCTTGAGGCAATCGTTTTTTTGATCGCATCGACGATGCTCGGATAGGATTCCACTATGGTATTCCGCAAGATAATGGTGTCGGGATTCAACGTTTTCAAGATGTTGAGAAGCCCGATGCCGATATGAAAGCCAAAGCGTTCAAACGTCTCCATCATGCCCGGATCACCGCGGTCTGCAAGTTCTTTAACGGTTTCGTAAAGCTGGGCCCCGGAATTCGCTGCATAGTGAGAGAAGACGGCTTTTTCTGAAGCGTAAAGCTCCCAGCAGCCTCTATTTCCGCACCGGCATAACGGACCGTCAAAGTGAATGGACATGTGCCCGGCTTCTCCGGAAAAACCTTGCACACCTCTAAACAATTTTCCATTCATTAAAATCCCTAAACCGATCCCGGTATTAATGCTGACAAACACGGCATGCTCAAGCTGGCCGCCTTCCCCGTATTCCTTCTCGCCAACGGCGCCGGCGTTGGCTTCGTTTTCAATTAAAATGGGAACGCCGAACCTTTCTTCCAGTTTTTCTTTTATCGGAATCAAATGGATCGGCTTATTTGGCGTAAAGACTACATGCCGTTCGTTGTCCACCAAGCCGGGCACGCACACGCCGATTCCGGTCAATCCGAATGGGGAAGGCGGTATTTTGTCAACGGCAAGCCCCGTCAATTCGATTAATGCCTCTTCTGTCGCCTGAATGTCTTCTTCATCAAGCGTTCGTTCGAATTGTTCAATCAAATGCCCTTCAAGGTCGGTTAACGCAACAATTATATAATTGGTTCCGACATCTACACCGACCGCATAGCCGGCTTTTCGATTGAATTTCAGCATAACGGGCCTTCTGCCGCCGCTTGACTCGCCGGGGCCGGTTTCATAAATGATGTCCTTTTGAAGCAGGGAAGACACTTGGGAGGAAACGGTCGCTTTATTTAATCCTGTTATTTCCGAAAGTTTGGCCCTTGAGACGGGTCCGTTTTCAATAATCTGCTCAAAAATCAGGGCTTTGTTCATTTTTTTTACAAGGGCTTGATCCGCTGTATTCAATGTCAATCACTCCATTGCTTTGAAGCTGTGAATTTATTATAGTATAACAAATTTTGAAAACTTATTTTTTCCTCTATTTCCATTGAAAGCGATTAATTGATCCTGTAAAATACATACAAGGAAGTTAGTTTAATGGTTAAACAAACATTGTTTTTTAACGTTTGCAAGGAAAAGTGAAGGGGGAGATCGGA |

Table S4. Summary of γ-PGA molecular weights in different production strains.

| **Strain** | **MWs (×10^5^Da)** | **γ-PGA yields (g/L)** | **reference** |
| --- | --- | --- | --- |
| *Bacillus amyloliquefaciens* NB | 0.2-0.3 | 17.62 | Sha et al., 2018 |
| *Bacillus subtilis* NX-2 | 0.2-10.0 | 25 | Yao et al., 2009 |
| *Bacillus licheniformis* PYA | 6.0-8.0 | 20.16 | Tian et al., 2013 |
| *Bacillus licheniformis* TISTR 1010 | 6.0-13.5 | 39.9 | Kongkelom et al., 2016 |
| *Bacillus subtilis* (chungkookjang) | 7.3-79.4 | ≤3.0 | Shih et al., 2005 |
| *Corynebacterium glutamicum* PGA011 | 20-40 | 21.3 | Xu et al., 2019 |
| *Bacillus licheniformis* ATCC 9945a | 3.18* | 34.93 | Feng et al., 2017 |
| *Bacillus amyloliquefaciens* NK-PV | 3.76* | 5.12 | Feng et al., 2014 |
| *Bacillus amyloliquefaciens* NK-pc | 4.14* | 7.12 | Feng et al., 2014 |
| *Bacillus subtilis* PB5522 | 14.5* | >40 | Scoffone et al., 2006 |

*means the values presented are the average molecular weights of PGA in each article.


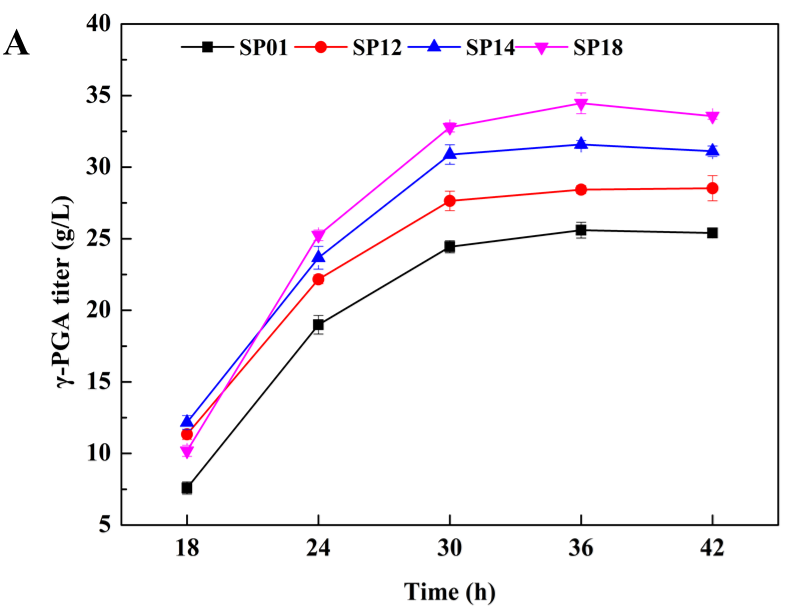

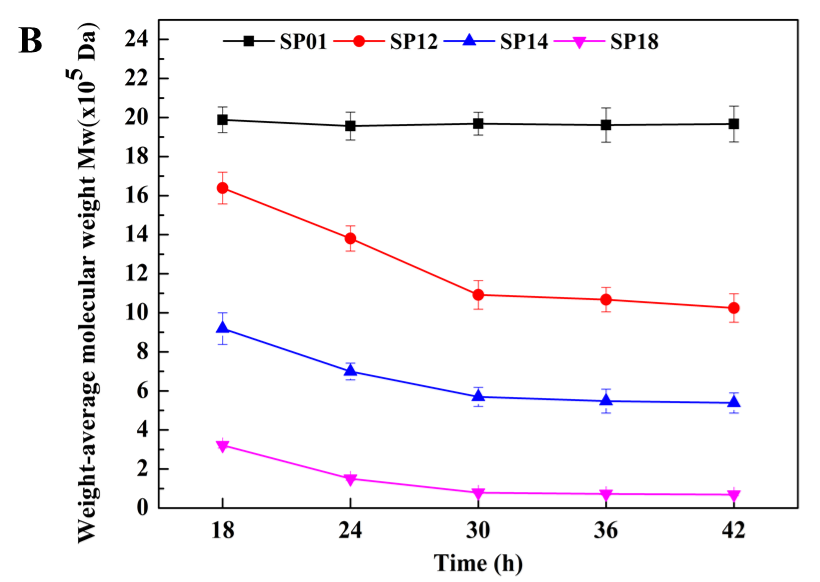


**Figure S1 Fermentation process curve of recombinant strains.**

(A) The γ-PGA titers of recombinant strains;

(B) weight-average molecular weight of γ-PGA at different times.


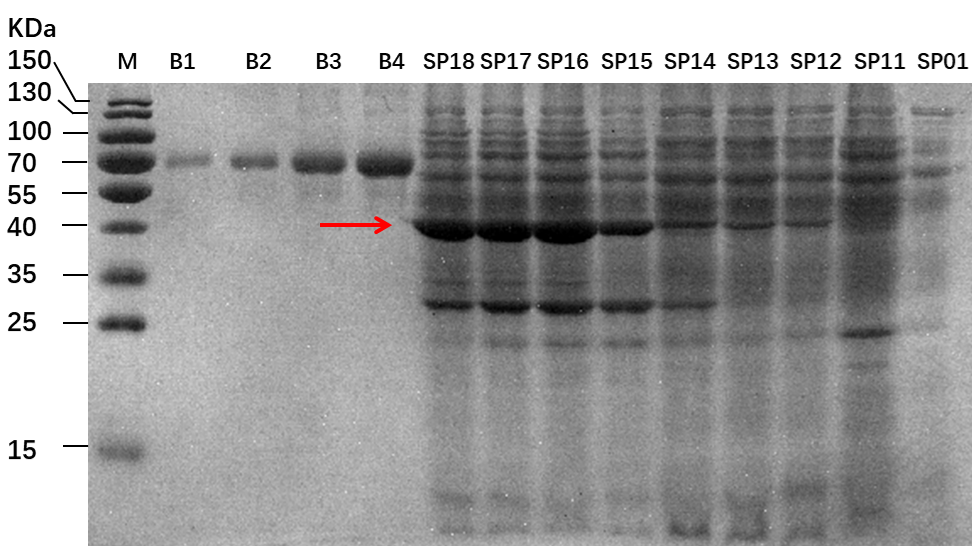


**Figure S2 SDS-PAGE analysis of the extracellular PgdS in recombinant strains with different promoters and signal peptides**. *M*: protein marker; B1: 0.1 g/L BSA; B2: 0.2 g/L BSA; B3: 0.4 g/L BSA; B4: 0.5 g/L BSA. SDS-PAGE was carried out in 1.0 mm thick 15 teeth gels. 10 uL of each samples contain loading buffer were loaded in the gel.


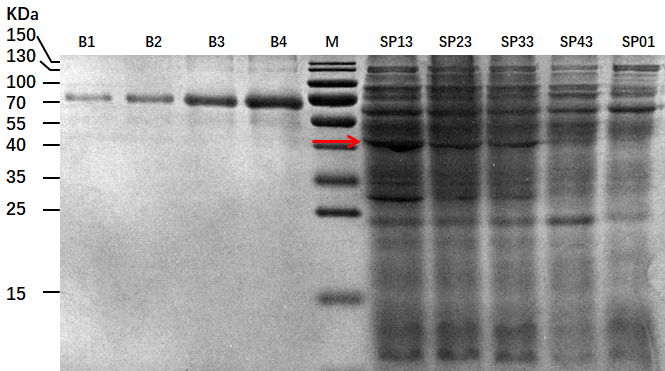


**Figure S3 SDS-PAGE analysis of the extracellular PgdS in recombinant strains with different promoters and signal peptides**. *M*: protein marker; B1: 0.1 g/L BSA; B2: 0.2 g/L BSA; B3: 0.4 g/L BSA; B4: 0.5 g/L BSA. SDS-PAGE was carried out in 1.5 mm thick 10 teeth gels. 20 uL of each samples contain loading buffer were loaded in the gel.


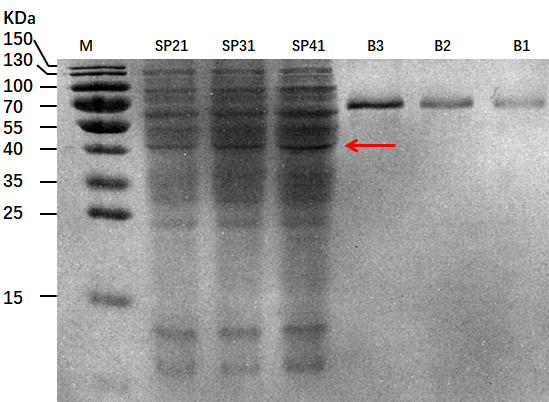


**Figure S4 SDS-PAGE analysis of the extracellular PgdS in recombinant strains with different promoters and signal peptides**. *M*: protein marker; B1: 0.1 g/L BSA; B2: 0.2 g/L BSA; B3: 0.4 g/L BSA. SDS-PAGE was carried out in 1.5 mm thick 10 teeth gels. 20 uL of each samples contain loading buffer were loaded in the gel.


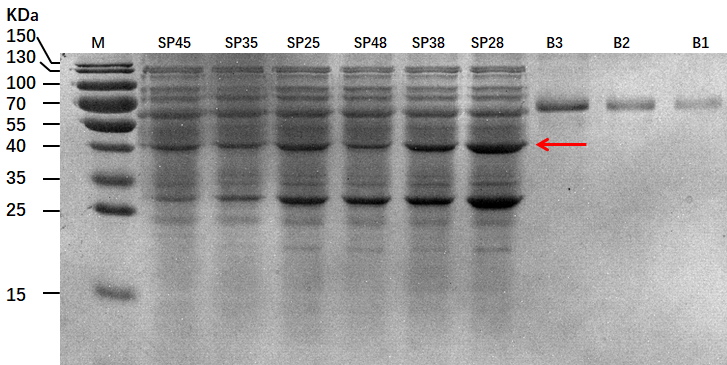


**Figure S5 SDS-PAGE analysis of the extracellular PgdS in recombinant strains with different promoters and signal peptides**. *M*: protein marker; B1: 0.1 g/L BSA; B2: 0.2 g/L BSA; B3: 0.4 g/L BSA. SDS-PAGE was carried out in 1.5 mm thick 10 teeth gels. 20 uL of each samples contain loading buffer were loaded in the gel.


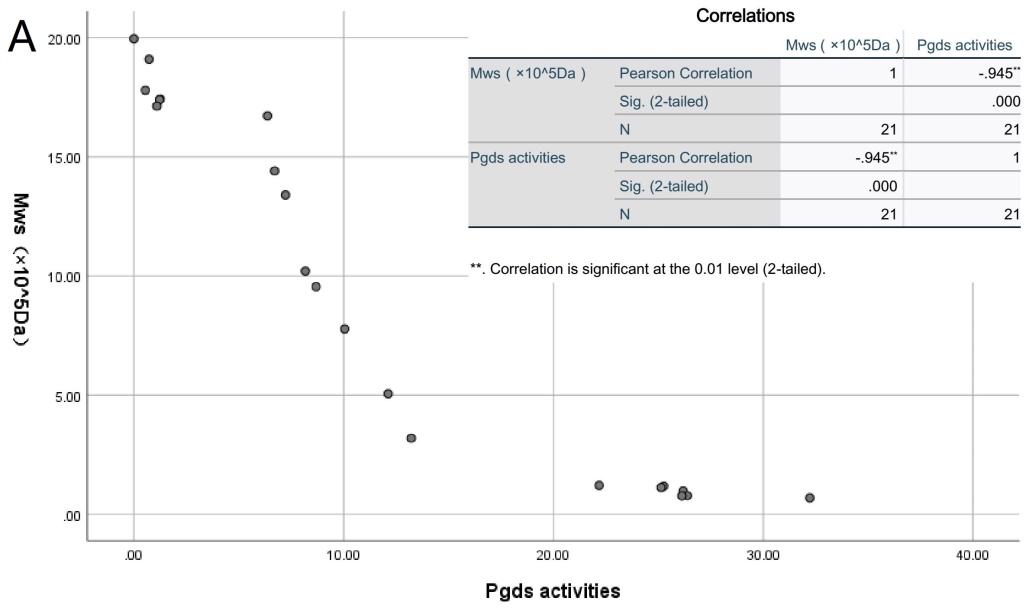


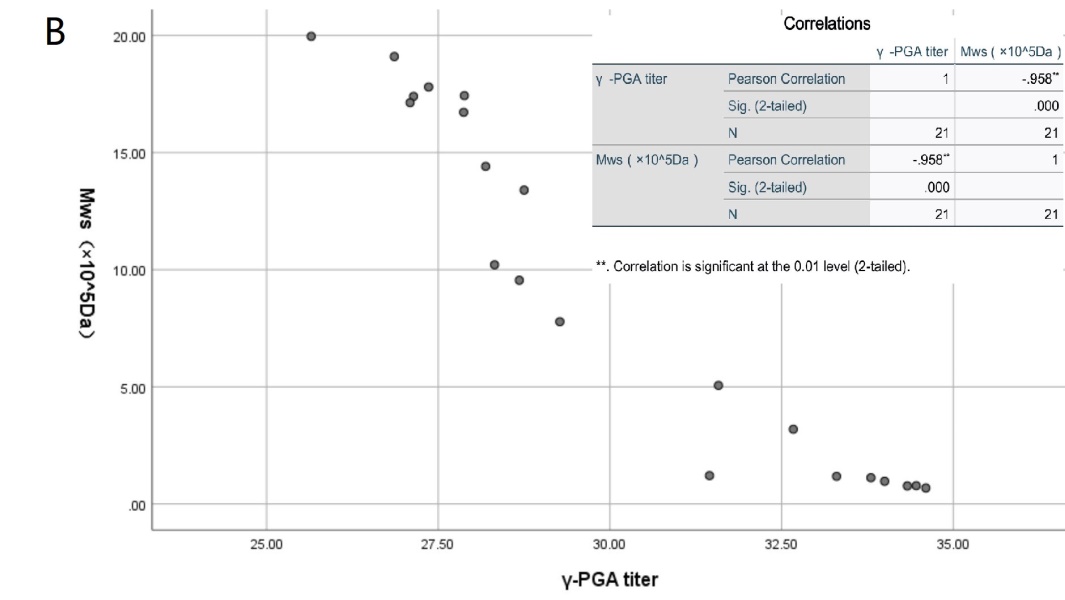


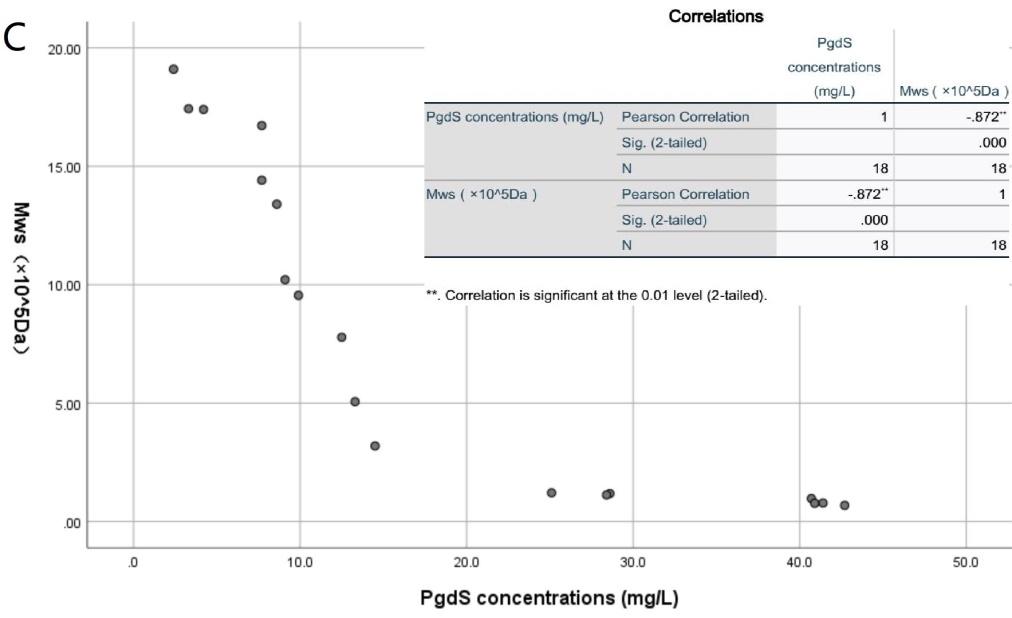


**Figure S6 Dispersion diagrams of molecular weights/PgdS activities (A), molecular weights/γ-PGA titer (B) and molecular weights/PgdS concentrations (C).** The Pearson’s value was R = -0.945, -0.958 and -0.872 with a bilateral signification of 0.01 (p <0 .01), respectively.
